# Dexmedetomidine inhibits abnormal muscle hypertrophy of myofascial trigger points *via* TNF-α/ NF-κB signaling pathway in rats

**DOI:** 10.3389/fphar.2022.1031804

**Published:** 2022-11-03

**Authors:** Mingjian Liu, Yu Liu, Xuan Li, Miao Pei, Mei Han, Feng Qi

**Affiliations:** ^1^ Department of Anesthesiology and Pain Clinic, Qilu Hospital, Cheeloo College of Medicine, Shandong University, Jinan, China; ^2^ Laboratory of Basic Medical Sciences, Qilu Hospital, Shandong University, Jinan, Shandong, China; ^3^ Department of Anesthesiology Clinic, Clinical Medical College & Affiliated Hospital of Chengdu University, Chengdu, China; ^4^ Department of the Quality Management, The Second Hospital of Shandong University, Jinan, China

**Keywords:** inflammation, inflammatory hypertrophy, myocyte enhancer factor 2C, chronic pain, myofascial pain syndrome

## Abstract

Myofascial pain syndrome (MPS) is a chronic pain disorder with inflammation-related primarily characterized by the presence of myofascial trigger points (MTrPs). Myocyte enhancer factor 2C (MEF2C) is involved in the occurrence of a variety of skeletal muscle diseases. However, it is not yet clear if MEF2C is involved in MTrPs. The purpose of this study was to investigate whether MEF2C was involved in the inflammatory pathogenesis of MTrPs. In the present study, we used RNA sequencing (RNA-seq) to compare the differential expression of myocyte enhancer factor 2C (MEF2C) in healthy participants and MTrPs participants. The widely used rat MTrPs model was established to research the upstream and downstream regulatory mechanism of MEF2C and found that MEF2C was significantly increased in patients with MTrPs. Dexmedetomidine (Dex) was injected intramuscularly in the MTrPs animal to assess its effects on MEF2C. The expression of MEF2C protein and mRNA in skeletal muscle of rats in the MTrPs group were up-regulated. In addition, the expression of TNF- α, p-P65, MLCK, and Myocilin (MyoC) was up-regulated and the mechanical pain threshold was decreased. Peripheral TNF- *α* injection significantly decreased the mechanical pain threshold and increased the expression of p-P65, MLCK, MEF2C, and MyoC in healthy rats. Maslinic acid increased the mechanical pain threshold and inhibited the expression of p-P65, MLCK, MEF2C, and MyoC. In addition, peripheral injection of DEX in MTrPs rats also inhibited the expression of TNF- α, p-P65, MLCK, MEF2C, and MyoC. These results suggest that MEF2C is involved in the inflammatory pathogenesis of MTrPs and DEX serves as a potential therapeutic strategy for the treatment of MPS.

## Introduction

Myofascial pain syndrome (MPS) is a chronic pain condition which is reported to affect approximately 85% of the population during their lifetime (Staud 2007). MPS is defined as a common clinical pain disorder primarily characterized by myofascial trigger points (MTrPs) (Alvarez and Rockwell 2002; Fede et al., 2020). The presence of taut bands and hyperalgesia in skeletal muscles and/or local twitch responses when conducting palpation are two of the most remarkable characteristics of MTrPs(Donnelly et al., 2019; Simons et al., 2019). Myofascial trigger points can be clinically classified as latent MTrPs or active MTrPs, according to whether spontaneous pain is caused (Bordoni et al., 2022). Myofascial trigger points not only induce pain directly, and can lead to autonomic nerve phenomenon, such as changes in skin temperature, sweating, tearing, and other autonomous nerve responses (Morikawa et al., 2017). The pathophysiological mechanism of MTrPs, however, is not entirely clear. The formation of MTrPs is a complex process of neuromuscular dysfunction, with various pathophysiological factors being involved in the initiation and progression (Stecco et al., 2013). In our previous studies, we used disposable biopsy instrument (SuperCore™, Argon Medical Devices, Inc.) for MTrPs muscle tissue and performed morphological observations. Hematoxylin and eosin (HE) staining revealed abnormal hypertrophy of skeletal muscle fiber at the MTrPs(Zhang et al., 2020b; Jin et al., 2020; Jin et al., 2020; Zhu et al., 2020). The muscle fibers in the MTrPs showed annular or elliptical muscle fibers of different sizes in cross-sectional views (Jin et al., 2020). Nevertheless, the molecular mechanisms underlying skeletal muscle fiber at the MTrPs hypertrophy are uncertain. In this study, we performed transcriptome sequencing analyses with human MTrPs and normal muscle tissue. Myocyte enhancer factor 2C (MEF2C) belongs to the MADS-BOX family of transcription factors and is considered to involve in skeletal muscle differentiation and growth (Gossett et al., 1989; Piasecka et al., 2021). Myocyte enhancer factor 2C activity is essential for pathological myocardial hypertrophy (Zhao et al., 2021), and inhibition of MEF2C has a protective effect on chondrocytes hypertrophy (Zhang et al., 2020). Moreover, MEF2C participates in the process of skeletal muscle atrophy and hypertrophic process in the soleus muscle after mechanical overloading (Sakuma et al., 2008; Judge et al., 2020).

Many previous studies proved that the histological hallmark of MTrPs is the abnormal hypertrophy of local skeletal muscle (Huang et al., 2015; Zhang et al., 2020b; Jin et al., 2020; Zhu et al., 2020). However, whether MEF2C is involved in MTrPs remain unknown. Many studies (Shah et al., 2008) support the argument that chronic aseptic inflammation may be involved in MTrPs. Recent studies have revealed that inhibition of miR-218-5p can suppress inflammation in myocardial ischemia-reperfusion injury *via* targeting MEF2C/NF-κB axis (Yang et al., 2021). And lipopolysaccharide (LPS) increases the activity of MEF2C *via* phosphorylates p38 to contribute to inflammation (Han et al., 1997). TNF- α, as a classical inflammatory factor, has been shown to increase significantly in MTrPs. (Shah et al., 2005). Meanwhile, TNF-α has been considered to be a key molecule in the regulation of myogenesis and skeletal muscle regeneration through its activation of p38 and MEF2C(Chen et al., 2007). These findings illustrate that may exist a positive feedback loop between TNF-α and MEF2C. In the process of skeletal muscle forming the MTrPs, it is unclear whether this regulatory pathway might exist. Therefore, we hypothesize that inflammation factors TNF-α might promote abnormal hypertrophy of muscle fibers by activating MEF2C. A human study and animal model experiment of MTrPs were conducted to test our hypothesis.

## Materials and methods

### Participants

This study was approved by the Human Research Ethics Committee of Qilu Hospital (KYLL-2014-027) and registered in the Chinese Clinical Trial Registry (ChiCTR-CPR-15007329). Written informed consent was obtained from participants before the study began.

The procedure used to recruit MPS participants (M group) and healthy controls (C group) was described in previous publications (Jin et al., 2020). A total of 18 participants who were diagnosed with myofascial pain syndrome by the Department of Orthopedics at Shandong University’s Qilu Hospital more than 3 months before the start of the study were enrolled. The MPS diagnosis and biopsy procedure of MTrPs were performed by the same physician in order to avoid any differences caused by different physicians. The diagnosis was made based on the Delphi study (Fernández-de-Las-Peñas and Jan, 2018; Gerwin, 2018), including an inquiry into the history of the disease and a physical examination. Participants in this study were included if they met the following criteria: 1) 18 - 65 years old. 2) any gender. 3) The upper trapezius muscle was diagnosed with MPS. The exclusion criteria: recent trauma, pregnancy, arthritis, diabetes, neurological disease, multiple sclerosis, and inflammatory diseases. The MTrPs group consisted of seven participants, while the control group consisted of five participants. Based on their medical histories, none of the participants had radiation, chemotherapy, or cervical radiculopathy.

### MTrPs biopsy procedure

As described in our previous publication (Jin et al., 2020), the procedure for sample biopsy is detailed. Briefly, biopsies from the MTrPs were obtained using a disposable SuperCore™ Biopsy instrument (ARGON) according to the international consensus on diagnostic criteria established in 2018 (Fernández-de-Las-Peñas and Jan, 2018; Gerwin 2018). The tissues were rapidly frozen in liquid nitrogen and stored at −80°C.

### RNA-seq

In this project, RNeasyMiniKit (Qiagen) was used to extract RNA from human upper trapezius muscle MTrPs samples. According to the TruSeq™ sample preparation guidelines (Chen et al., 2015), the TruSeq^TM^RNA sample preparation kit was used to synthesize a pair-end library. We use the concept of FPKM to express the expression of different genes, use edgeR software for statistical quantification, calculate the number of sequencing reads of each gene and homogenize between human upper trapezius samples, on this basis, according to the experimental grouping information to calculate the differences between the two groups of genes. The gene fold change difference between groups was calculated according to the FPKM value.

### MTrPs animal model and measurement of mechanical hyperalgesia.

Shandong University’s Animal Care and Use Committee approved all animal experiments. Qilu Hospital of Shandong University Experimental Animals Laboratory provided 6-week-old male/female Sprague-Dawley (SD) rats weighing 200–250 g. In standard conditions (room temperature: 24°C; relative humidity: 20%–30%), rats were raised on a 12-h light/12-h dark cycle. In each cage, four rats were housed independently, and food and water were provided *ad libitum*. One week before the experiment, the rats were acclimated to the environment.

In the current study, we used the MPS animal model reported by Huang et al., which is the most widely used animal model of MPS(Li et al., 2019; Li et al., 2019; Zhang et al., 2020a; Ye et al., 2020). The diagnosis was done according to the standard diagnosis with MPS criteria based on the 2018 diagnosis (Fernández-de-Las-Peñas and Jan, 2018; Gerwin, 2018), including the presence of TBs and a hypersensitive spot (MTrPs). This animal model, a chronic pain animal model, well mimics the two characteristic features of MPS. Moreover, the muscle fibers in the MTrPs rat model are morphologically similar to that seen in human muscle biopsy. Therefore, this model is the most appropriate model for the study of MPS both behaviorally and histologically. The procedure of the animal model has been studied and exhaustively described in previous studies. Briefly, the left gastrocnemius muscle (GM) of rats was struck bluntly (2.352 J energy) and eccentric-based exercises (downward angle of -16, speed of 16 m/min, lasted for 1.5 h) were performed on the first and second day of each week for a total of 8 weeks. During the remaining 4 weeks, the diets were well fed with no other manipulations. The position of the MTrPs was determined by palpation of TBs and needling (LTRs). For mechanical withdrawal pain threshold, a Randall-Selitto instrument (Shandong Institute of Science and Technology) equipped with a round head probe (tip diameter: 8 mm) was used. Randall-Selitto tests involve increasing pressure until the rat withdraws its limb, the pressure value is automatically recorded. The withdrawal pain threshold was measured five times for each rat with an interval of 3 minutes, and the maximum and minimum values were removed for statistical analysis. The rats were randomly divided into eight groups using a web-based random number generator (GraphPad software): Control + dimethyl sulfoxide (DMSO) (*n* = 3), Control + TNF-α (Abcam, 0.1 mg/m, *n* = 6), Control + Maslinic acid (MCE, 5 mg/ml) + TNF-α (0.1 mg/ml) (*n* = 5), MTrPs + DMSO (*n* = 3), MTrPs + NF-κB inhibitor Maslinic acid (5 mg/ml, *n* = 6), MTrPs + Dexmedetomidine (10 μg/ml, *n* = 6), MTrPs + Dexmedetomidine (100 μg/ml, i. m.n = 6), MTrPs + Dexmedetomidine (100 μg/ml, i. v.n = 6).

### Hematoxylin-eosin staining

Control and MTrPs specimens were fixed using GD fixative solution (Sevicebio, Wuhan, China). In the following stages, specimens were dehydrated, paraffin-embedded, and sectioned. A 4-um thick section of tissue was attached to glass slides, dewaxed, and stained with HE. In the next step, the sections were dehydrated in ethanol (70–100%) and xylene, followed by cover slips. Slices were examined with a digital camera attached to an optical microscope.

### Immunofluorescence

Sections of paraffin-embedded tissue were heated at 65°C for 2 h, separated in xylene, and rehydrated in graded ethanol at room temperature. Triton X-100 was applied for 10 min after washing with PBS three times. Sections were washed three times in PBS and then microwaved with a sodium citrate buffer (PH = 6). A wet chamber was used to incubate the slices in 3% hydrogen peroxide for 10 min after three washes in PBS. The tissue sections were washed three times in PBS and treated for 30 min with normal goat serum at 37°C. At 4°C, muscle sections were then incubated overnight with primary antibody (MEF2C, Santa, sc-518152, 1:200). The next day, secondary antibodies were incubated at 37°C for 30 min with dye-labeled secondary antibodies (Abbkine, A23210). After three rounds of washing with PBS, tissue sections were treated with DAPI for 5 minutes and covered.

### qRT-PCR

In this experiment, Trizol (Beyotime, R0016) was used to extract total RNA from MTrPs and normal rat gastrocnemius muscle tissue. The total RNA was transcribed into cDNA by HiScript III RT SuperMix for qPCR (+gDNA wiper) kit (Vazyme, R323-01). cDNA was subsequently amplified by PCR with specific primers. ChamQ universal SYBR qPCR Master Mix (Vazyme, Q711-02) was used to perform quantitative real-time PCR. Levels of targeted mRNA were normalized to GAPDH. The primers are listed in table 1.

**TABLE 1:** The sequence of the primers used in the current investigation in RT-qPC.

Gene	Forward primer 5′→3′	Reverse primer 5′→3′
MLCK	GAG​GAT​CGT​GGA​TGA​GGA​CTA​CC	ACA​CAG​GAT​GTT​CTC​TGG​CTT​GA
MEF2C	GAG​GAT​GTG​GAC​TTG​CTG​TTG​AA	TGT​TGT​TGA​AAT​GGC​TGA​CGG​ATA
MyoC	AGG​TAG​CAA​GGC​TGA​GGA​GAG	CCA​AAT​TGG​ACT​GAG​AGA​CTT​CCC
GAPDH	TCT​CTG​CTC​CTC​CCT​GTT​CT	ATC​CGT​TCA​CAC​CGA​CCT​TC

### Western blotting

Liquid nitrogen was used to store the muscle tissues at the injection site of the rat’s left gastrocnemius. Lysis buffer (containing protease inhibitors and phosphatase inhibitors) was used with the tissue samples. Centrifuge the lysed homogenate at 14,000 rpm for 20 min at 4°C. The supernatants were collected and dissolved in 4°C sample buffer before being denatured at 100°C for 10 min. Afterward, the proteins were separated by SDS-PAGE and transferred to PVDF membranes, which were blocked for 1 h at room temperature with QuickBlock™ Blocking Buffer (Beyotime Biotechnology, P0252-500 ml), before incubating at 4°C overnight with the antibodies (TNF-α, Affinity, AF7014, 1:500; p-P65, Abcam, ab76302, 1:1000; P65, CST, 8242T, 1:1000; MLCK, Affinity, DF9023, 1:1000; MEF2C, Santa, sc-518152, 1:500; MyoC, Affinity, DF6483, 1:1000; GAPDH, Beyotime, AF1186, 1:10000; Lamin B1, Abcam, ab133741, 1:5,000). Membranes were washed with Tris-buffered saline Tween-20 (TBS-T) and incubated at room temperature for 1 h with secondary antibody (Bosterbio, BA1054, 1:10000; Bosterbio, BA1050, 1:10000). With a chemiluminescent reagent, the blots were developed.

### Statistical analysis

For data analysis, GraphPad Prism 8.0 statistical software was used. All variables were obtained from at least three repeat independent experiments unless otherwise stated, and are presented as mean +standard deviation (SD). Student’s t-test was used to analyze the differences between the two groups. The *t* test was used for data with normal distribution, and the Mann-Whitney *U* test was used for data that did not meet normal distribution. The main effects and interactions of two factors were determined using one-way ANOVA or two-way ANOVA, followed by Tukey’s multiple comparison test to determine differences between groups where necessary. Pain withdraw threshold data measured over time were analyzed using repeated measures ANOVA. A *p*-value smaller than 0.05 (*p* < 0.05) was considered statistically significant.

## Results

### MEF2C was found to be upregulated in human and animal MTrPs tissue and participated in the pathophysiological process of MTrPs

**FIGURE 1:**
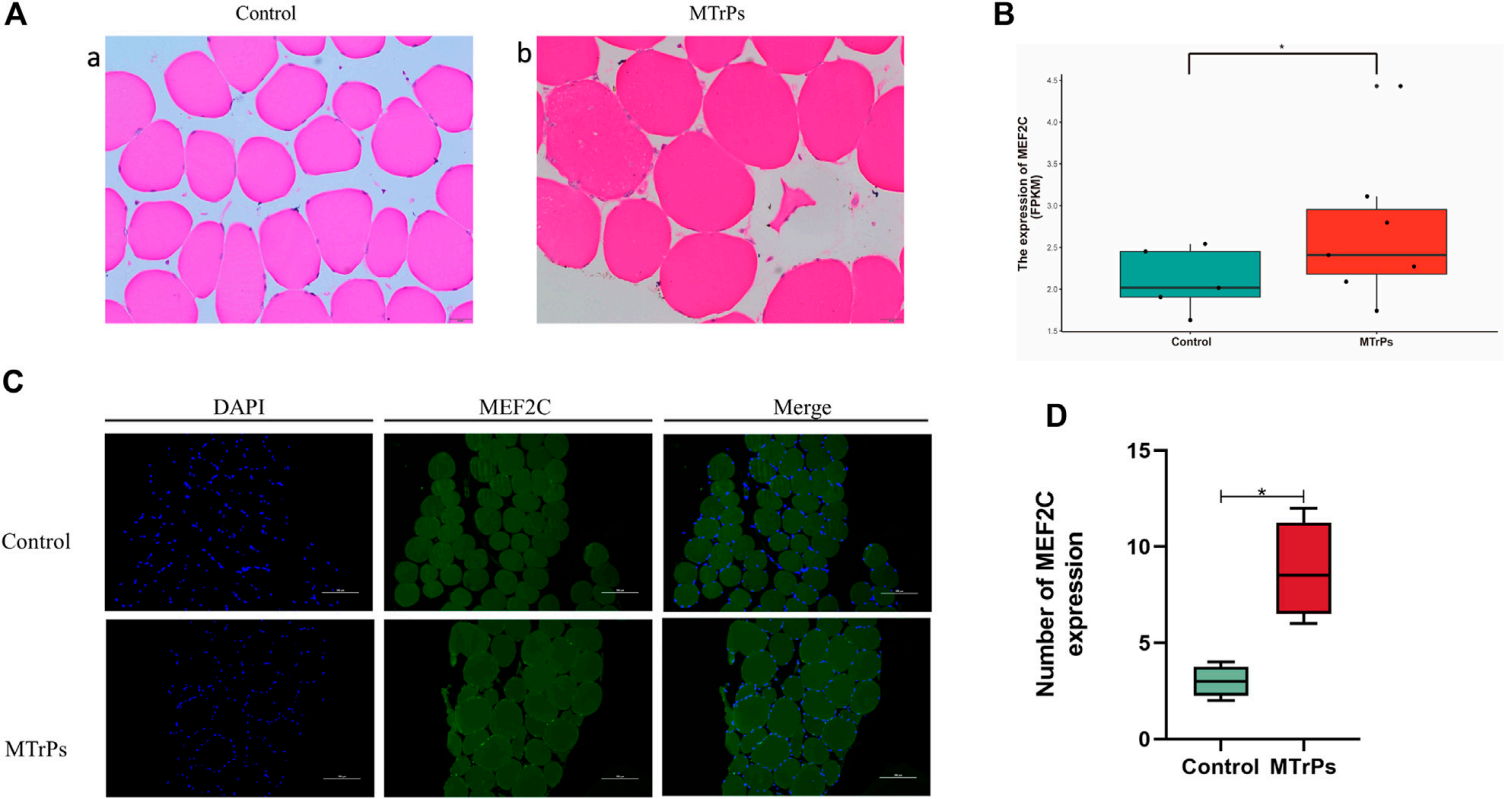
**(A)** H&E staining of the human trapezius muscle fibers cross-sections with light microscopy (x 400). (a) Control muscle fiber morphology. (b) Enlarged and round muscle fibers were observed in MTrPs patients. **(B)**. RNA-seq was used to compare the transcriptome differences of MEF2C between healthy human control group (*n* = 5) and MTrPs group (*n* = 7, log_2_FC = 10.20419, *versus* control using unpaired Mann-Whitney *U* Test, *p* < 0.05). **(C)**. Immunofluorescence staining of trapezius muscle fibers in human control and MTrPs groups, showed a significant increase in nuclear translocation in the MTrPs group. **(D)**. The number of MEF2C expression in the Control and MTrPs group. *p < 0.05, **p < 0.01.

Human trapezius tissue was taken for H&E staining and observed under the microscope. In the control group, the fibers were polygonal in shape (Figure 1A). However, the fibers of the MTrPs group were enlarged and round (Figure 1A). The RNA sequencing results showed that the expression of MEF2C genes was up-regulated in MTrPs tissue (Figure 1B). MEF2C is the second most highly transcribed gene in MTrPs tissue compared to normal tissue. Immunofluorescence for MEF2C showed a significant increase in nuclear translocation in human MTrPs groups (Figures 1C,D).

**FIGURE 2:**
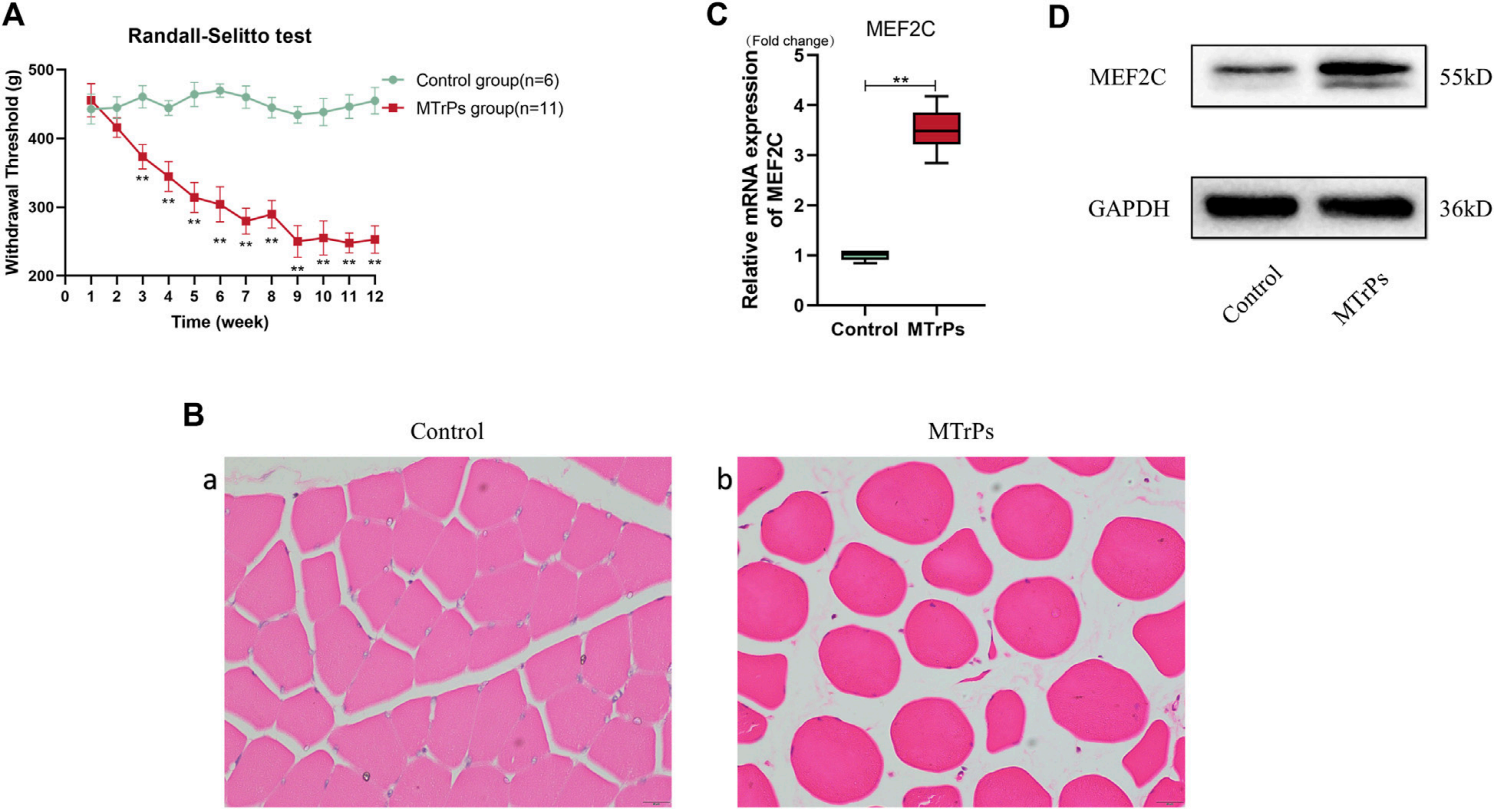
**(A)** Weekly changes in mechanical withdrawal thresholds in the left gastrocnemius muscle of rats were measured with the RandallSelitto device. The mechanical withdrawal threshold decreases from week three until it remains low at week 12. Data are presented as the mean values ±standard deviation. **(B)** H&E staining of the MTrPs model of rats muscle fibers cross-sections with light microscopy (x 400). (a) Normal muscle fibers have a uniform size and a polygonal shape. (b) Deeply stained round muscle fibers with central nucleus of variable size were seen in the MTrPs group. **(C)**. The mRNA expression of MEF2C in the MTrPs group was higher than that in the control group, using GAPDH as the internal reference mRNA. **(D)**. MEF2C protein expression was significantly higher in the MTrPs group, with GAPDH as the internal reference protein. *p < 0.05, **p < 0.01.

The animals model was established successfully verified by results of mechanical withdrawal thresholds and HE staining after a blunt strike on the left gastrocnemius muscles and eccentric exercises for 8 weeks subsequent 4 weeks of recovery (Figure 2A,B). In the animal MTrPs model, the transcript levels of MEF2C genes were increased by qRT-PCR experiments (Figure 2C). MEF2C was measured by Western blotting experiments, consistent with the qRT-PCR results (Figure 2D). A significant increase of MEF2C levels was observed, either in transcript or in protein expression levels. These results demonstrate that MEF2C is involved in the pathophysiological process of MTrPs. In many studies, MEF2C has been shown to play a critical role in muscle differentiation and maturation. Our initial results suggest that dysplastic aspects are evident in MTrPs and associated with MEF2C. To clarify the regulatory relationship of MTrPs muscle development associated with MEF2C, we conducted a series of experiments.

### Overexpression of TNF-α induced activation of NF-κB p65, MLCK, and MEF2C and causes mechanical hyperalgesia when injected gastrocnemius muscles *in vivo*

**FIGURE 3:**
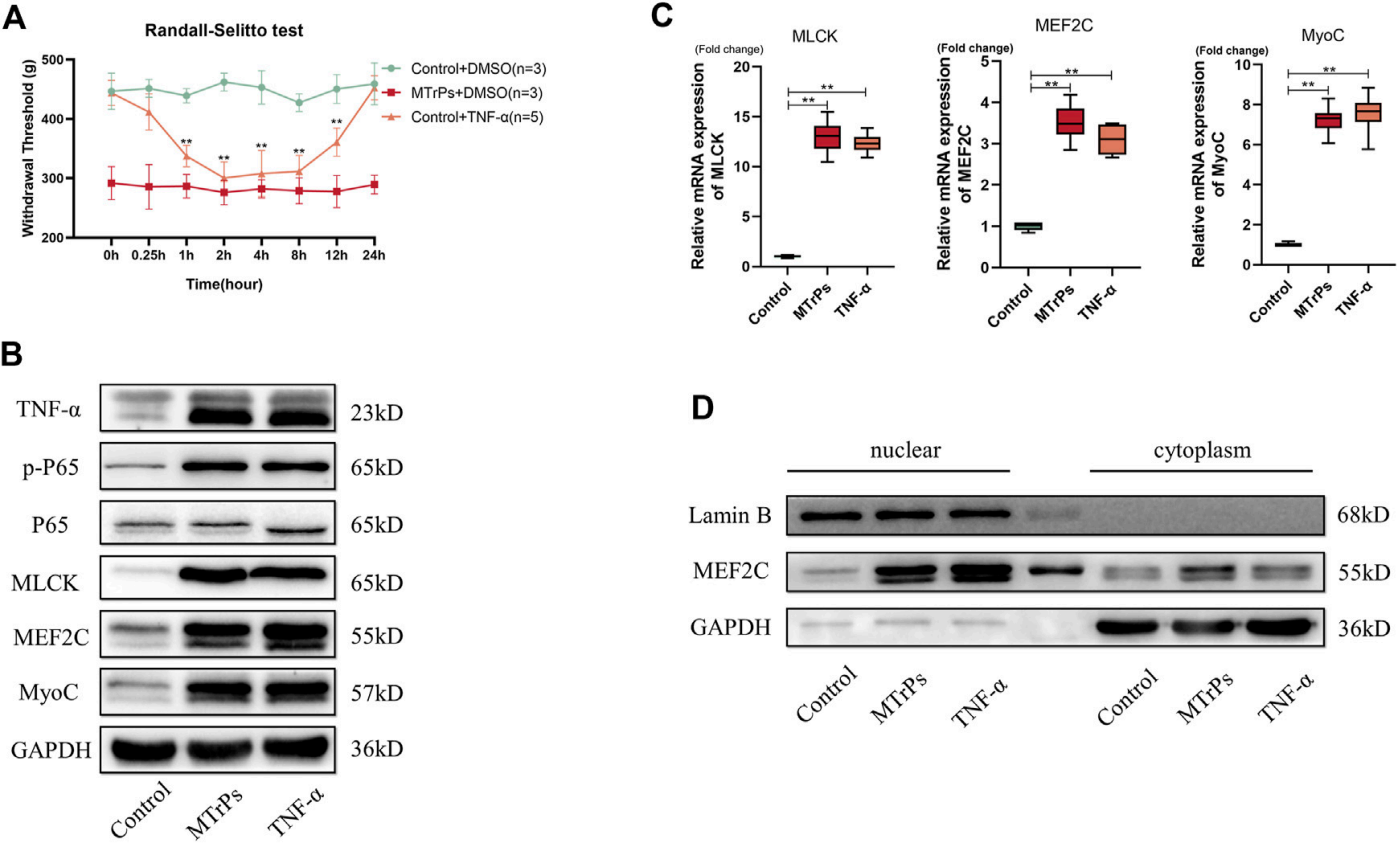
**(A)** Effect of TNF-α on mechanical withdrawal threshold in control group rats. Decreased mechanical withdraw threshold in control rats (*n* = 5) at 1, 2, 4, 8, 12 h after intramuscular injection of TNF-α (0.1 mg/ml). **(B)**. TNF-α (0.1 mg/ml) could induce the high expressions of p-P65, MLCK, MEF2C, and MyoC. Expression levels were detected 2 h after injection of TNF-α or DMSO and the differences were statistically significant. **(C)**. The mRNA expression levels of MLCK, MEF2C, and MyoC were higher in the MTrPs group and TNF-α group than in the control group, with GAPDH as the internal reference mRNA. **(D)**. Using GAPDH as cytoplasmic protein internal reference and Lamin B as cytosolic protein internal reference, the nuclear protein expression of MEF2C was higher in both MTrPs and TNF-α groups than in the control group. *p < 0.05, **p < 0.01.

Our previously unpublished studies found that elevated expression of these inflammatory cytokines in MTrPs tissue, including TNF-α and IL-6R. Inflammation is not the end point of pathophysiological change, and the inflammatory response may play an essential role in the pathogenesis and process of MTrPs through other novel mechanisms or pathways. To confirm our hypothesis, we injected animals with recombinant TNF-α (0.1 mg/ml, 30 ul * 3 points) to gastrocnemius muscles in healthy rats. The rats developed mechanical hyperalgesia and withdrawal thresholds decreased following recombinant TNF-α injection (Figure 3A). This decreased mechanical withdrawal thresholds started 15 min after recombinant TNF-α injection, reached its lowest point at the 2 h after injection, and lasted for about 12 h. The rats were sacrificed, and the muscle was extracted at 2 h for Western blotting and qRT-PCR tests. The expression of the TNF-α and Phospho-NF-κB p65 in the MTrPs was significantly upregulated compared to that in the control group as measured through Western blotting (Figure 3B), consistent with the qRT-PCR results (Figure 3C). In addition to this, MLCK and MEF2C were upregulated in the MTrPs tissue and induced MEF2C nuclear translocation (Figure 3D). MyoC, as the downstream of MEF2C, was enhanced the expression (Figure 3C). In previous studies, MLCK was confirmed to directly phosphorylate MEF2C and exerted its biological functions by promoting its nuclear translocation.

Peripheral injection of TNF-α induces the overexpression of a subset of NF-κB target genes, including MLCK, MEF2C, and MyoC. These results suggest that TNF-α injected into the peripheral gastrocnemius muscles may activate signaling pathways of abnormal muscle development, thereby inducing abnormal muscle hypertrophy and producing nociception.

Inhibition of phosphorylation of NF-κB p65 suppressed activation of MEF2C and reversed the pain behaviors induced by MTrPs.

**FIGURE 4:**
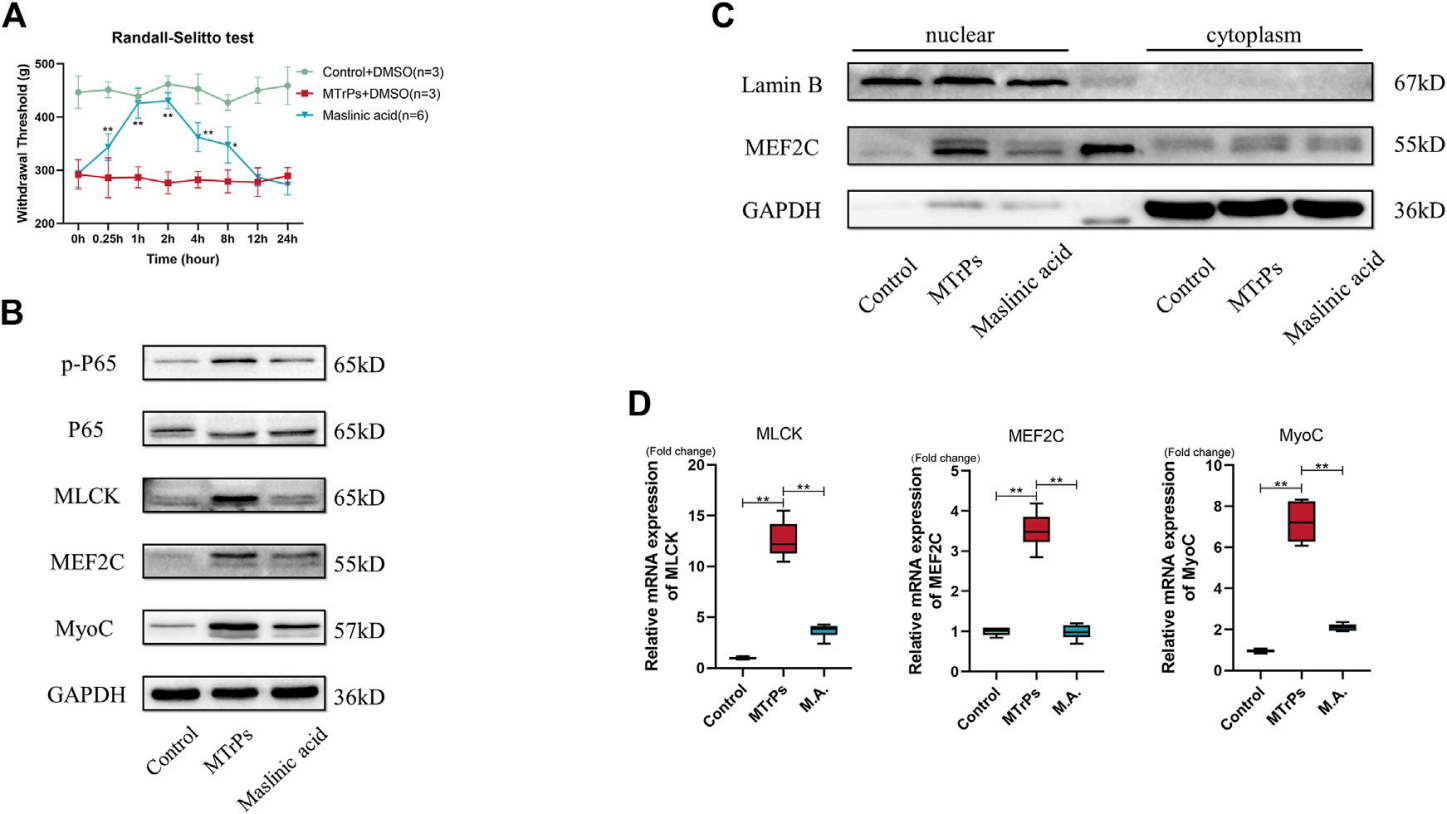
**(A)** Effect of Maslinic acid on mechanical withdrawal thresholds in MTrPs rats (*n* = 5). M.A. (5 mg/ml) after intramuscular injection at 0.25, 1, 2, 4, and 8 h rescued the decrease of mechanical withdrawal threshold in MTrPs rats. **(B)**. Expression of p-P65, P65, MLCK, MEF2C, and MyoC was detected 1 h after M.A. or DMSO injection, and the protein expression of p-P65, MLCK, MEF2C, and MyoC in the M.A. group was significantly lower than that in the MTrPs group. **(C)**. The nuclear protein expression of MEF2C in the M.A. group was lower than that in the control group, with GAPDH as the cytoplasmic protein internal reference and Lamin B as the cytosolic protein internal reference. **(D)**. The mRNA expression levels of MLCK, MEF2C, and MyoC in the M.A. group were significantly lower than those in the MTrPs group, using GAPDH as the internal reference mRNA.

Our study showed that may be NF-κB involved in MTrPs muscle hypertrophy. Therefore, inhibition of phosphorylation of NF-κB p65 (Maslinic acid, 5 mg/ml, 30 ul * three points) was injected intramuscularly and mechanical withdrawal thresholds were measured in the MTrPs group 0.25, 0.5, 1, 2, 4, 8, 12 and 24 h after muscle injection, and the analgesic effect of Maslinic acid reached its peak by 1 h (Figure 4A). The MTrPs tissue was extracted at 1 h for Western blotting aND qRT-PCR tests. Western blotting showed that the phosphorylation level of NF-κB p65, the expression of MLCK and MEF2C was downregulated, and the expression of MyoC downstream was also inhibited compared with MTrPs + DMSO group (Figure 4B). In addition, Maslinic acid inhibits TNF-induced MEF2C nuclear translocation (Figure 4C). The mRNA expression of MLCK, MEF2C, and MyoC were consistent with the Western blotting results (Figure 4D).

**FIGURE 5:**
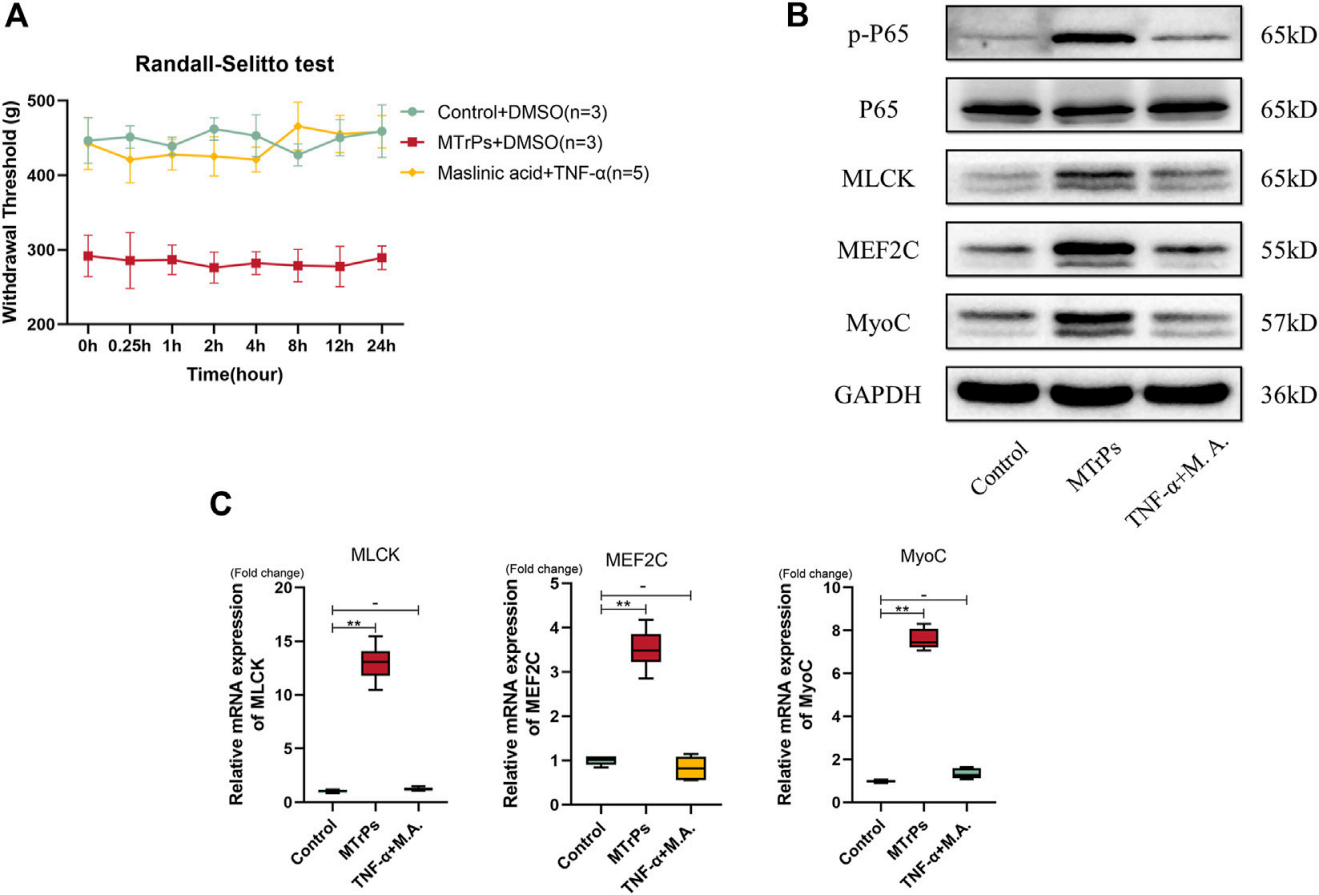
**(A)** The changes in mechanical pressure pain thresholds in the Control group of rats injected with Maslinic acid (5 mg/ml) 1 h in advance followed by TNF-α (0.1 mg/ml) were not statistically different from the control group. **(B)**. The expression of p-P65, P65, MLCK, MEF2C, and MyoC was detected 2 h after injection of M.A. and TNF-α. There was no statistical difference in expression levels between the two groups. **(C)**. The mRNA expression levels of MLCK, MEF2C, and MyoC in the M.A. and TNF-α injection groups were not significantly different, and GAPDH was used as the internal reference mRNA. *p < 0.05, **p < 0.01.

To prove that peripheral TNF-α mediated peripheral pain sensitization and abnormal muscle hypertrophy *via* the NF-κB pathway, a rescue experiment was performed on healthy rats. After Maslinic acid (5 mg/ml, 30 ul * three points) intramuscular injection 1h, we made a recombinant TNF-α (0.1 mg/ml, 30 ul * three points) injection successively. The decrease in mechanical withdrawal thresholds induced by recombinant TNF-α was partially reversed by the administration of maslinic acid (Figure 5A). MLCK, MEF2C, and MyoC expressions were rescued by Maslinic acid injection (Figure 5B). The mRNA expression of MLCK, MEF2C, and MyoC were consistent with the Western blotting results (Figure 5C). These findings suggest that peripheral TNF-α was involved in the peripheral pain sensitization and abnormal muscle hypertrophy induced by MTrPs through the NF-κB pathway.

### Peripheral injection of dexmedetomidine inhibited NF-κB p65, MLCK, MEF2C, and MyoC expression, which were upregulated in MTrPs

**FIGURE 6:**
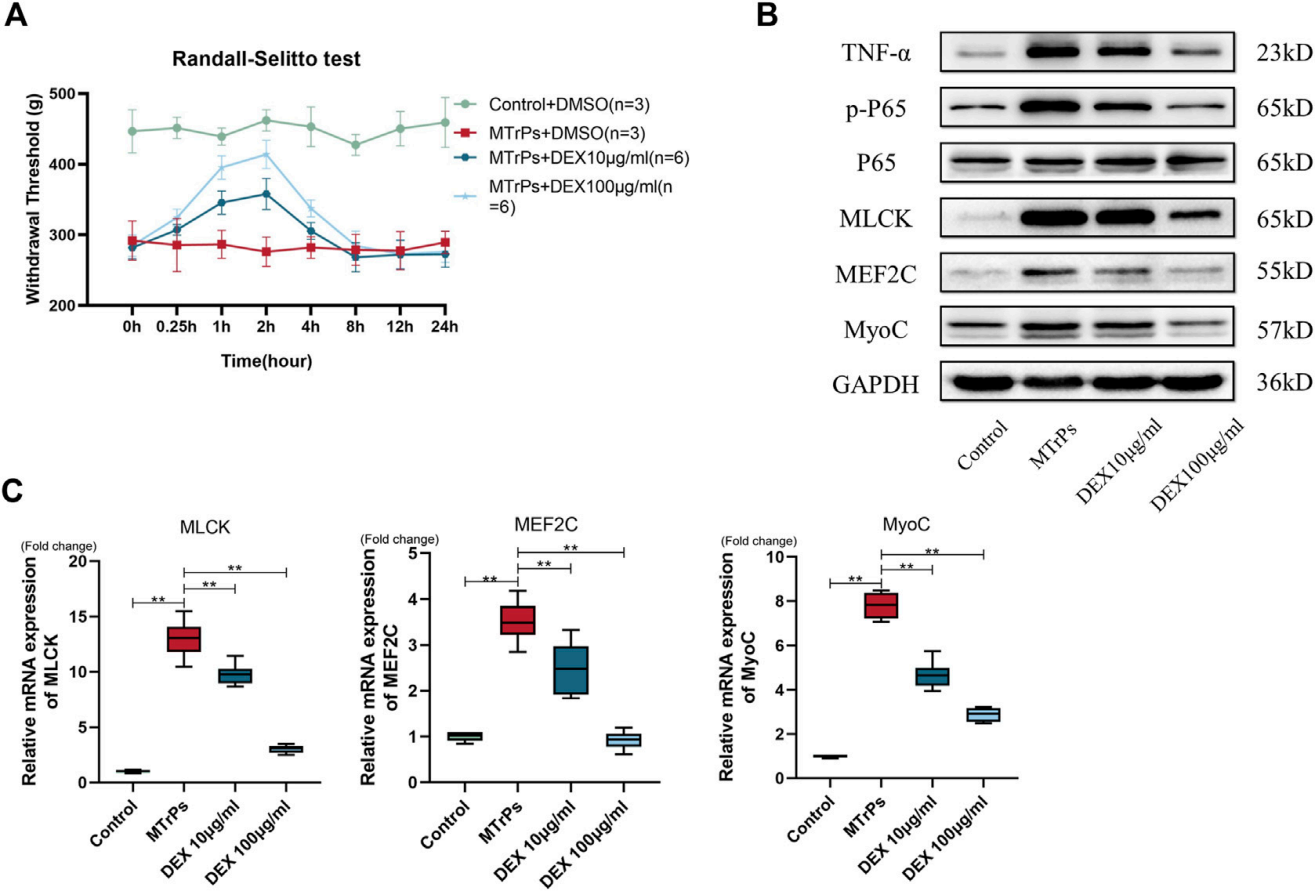
**(A)** Effect of DEX on mechanical withdrawal threshold in MTrPs group rats. Increased mechanical withdrawal threshold in MTrPs rats (*n* = 6) at 1 and 2 h after intramuscular injection two concentrations of DEX (10ug/ml and 100ug/ml). **(B)**. The expression of TNF-α, p-P65, P65, MLCK, MEF2C, and MyoC was detected 2 h after injection of two different concentrations of dexmedetomidine (10 μg/ml and 100 μg/ml) into the gastrocnemius muscle of MTrPs rats, and the protein levels were found to be significantly inhibited. **(C)**. The mRNA expression levels of MLCK, MEF2C, and MyoC were significantly lower than those of the MTrPs group after dextromethorphan injection, with GAPDH as the internal reference mRNA. *p < 0.05, **p < 0.01.

Two concentrations of DEX (10 μg/ml and 100 μg/ml, 30 μl * three points) were injected into peripheral gastrocnemius muscles in MTrPs animals (*n* = 12, two groups, six per group). And mechanical withdrawal thresholds were measured at 0.25, 0.5, 1, 2, 4, 8, 12 and 24 h after injection. and the study results showed that the analgesic effect of DEX reached its peak by 2 h (Figure 6A). After injection of 2 h, the MTrPs rats were killed and tissue was flash frozen in liquid nitrogen for tests. The expressions of TNF-α, Phospho-NF-κB p65, MLCK, MEF2C, and MyoC proteins in the MTrPs group were decreased dose-dependently compared to the MTrPs group (Figure 6B). The mRNA level regulation followed the same trend as the protein level identified from Western blotting described above (Figure 6C). These results further illustrated that DEX has the ability to inhibit hyperpathia and abnormal muscle hypertrophy mediated by TNF-α/NF-κB pathway.

### DEX acted locally in the MTrPs microenvironment, and not systemically

**FIGURE 7:**
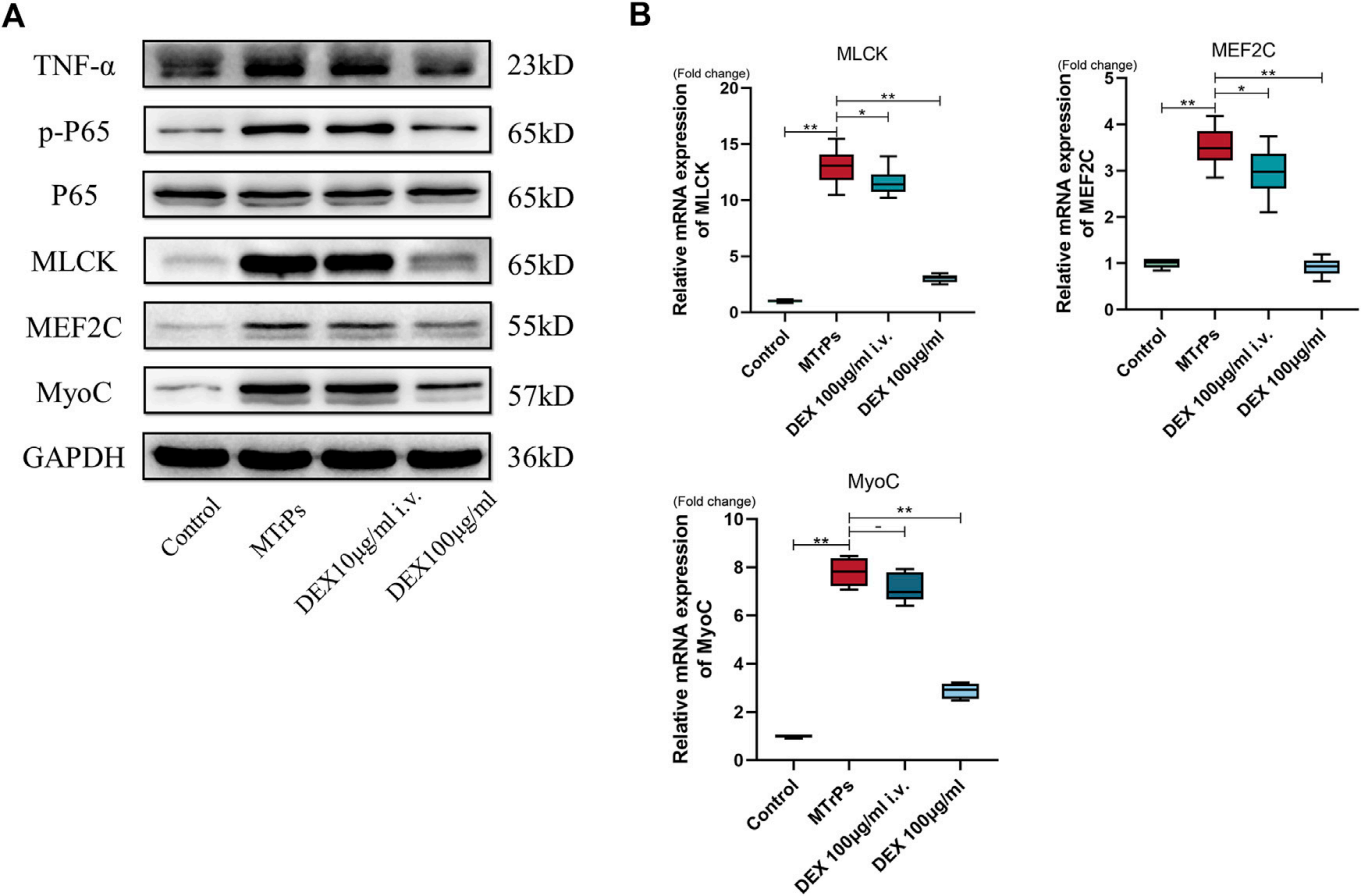
**(A)** The expression of TNF-α, p-P65, P65, MLCK, MEF2C, and MyoC was detected in MTrPs rats after 2 h of tail vein injection and local gastrocnemius injection with the same concentration of dexmedetomidine (100 μg/ml), and it was found that the protein levels in the gastrocnemius group were significantly lower than those in the MTrPs and intravenous injection groups. **(B)**. The mRNA expression levels of MLCK, MEF2C, and MyoC in the local injection of the dexmedetomidine group were lower than those in the MTrPs and intravenous injection groups, with GAPDH as the internal reference mRNA. *p < 0.05, **p < 0.01.

To test whether the same effect occurs in systemic administration, DEX (100 ug/ml) was injected to two MTrPs group rats (*n* = 12, six per group) *via* the tail vein and peripheral gastrocnemius muscles separately. After administration for 2 h, samples were extracted for tests. The experimental results showed that the administration cannot decrease the expression levels of MLCK, Phospho-NF-kB p65, MEF2C, and MyoC *via* the tail vein (Figures 7A,B). Thus, the effects of DEX act on MTrPs microenvironment, instead of the central system.

## Discussion

In the present study, we found the following results. 1) Using SuperCore biopsy needle, trapezius samples were obtained from MTrPs patients and high throughput mRNA sequencing (mRNA-seq) was performed to compare the expression between patients with MTrPs and healthy controls. Sequencing results showed MEF2C was significantly higher in participants with MTrPs than in healthy controls. To validate the results obtained by sequencing, we performed MEF2C immunohistochemistry and immunofluorescence staining paraffin sections of human MTrPs tissue specimens. We found that the expression of MEF2C was increased and activated nuclear translocation. These findings illustrate that MEF2C may have participated in the formation of MTrPs. 2) Injection of recombinant TNF-α promoted MLCK, MEF2C, and MyoC expression *via* NF-κB p65 signaling pathway in healthy rats. This indicated that TNF-α can lead to abnormal muscle fibers hypertrophy. 3) Inhibition of NF-κB p65 reversed the pain behaviors and increased mechanical withdrawal thresholds induced by MTrPs. Moreover, the expression and nuclear translocation of MEF2C was also inhibited. This indicated that the NF-κB p65 signaling pathway was involved in peripheral pain sensitization and abnormal muscle fiber hypertrophy induced by MTrPs. 4) DEX can be acting at the peripheral level to attenuate inflammation and inhibit the NF-κB p65 signaling pathway activation and consequently reduces the expression of MEF2C. Thus suggesting that DEX might have clinical applications in the therapy of MTrPs/MPS.

MEF2C, a key transcription factor for muscle development, is the first DNA binding transcription factor activity known to have muscle properties that bind to muscle-specific gene promoters and regulate muscle development (Potthoff and Olson 2007; Mokalled et al., 2012; Liu et al., 2014). Skeletal muscle expression of MEF2C is essential to maintain the integrity and normal morphology of postnatal muscle fibers (Potthoff et al., 2007a; Potthoff et al., 2007b). Low expression of MEF2C has been confirmed to be associated with multiple muscle atrophy conditions, including cancer cachexia-induced skeletal muscle wasting (Shum et al., 2012; Judge et al., 2020). Although very few studies investigated the role of MyoC in skeletal muscle, it has been thoroughly established as a prohypertrophic protein that binds and stabilizes the dystrophin-glycoprotein complex (DGC), having the effects of stabilizing the muscle fiber membrane (Joe et al., 2012b). In addition, overexpression of MyoC leads to muscle hypertrophy by increasing the cross-sectional area and weight of skeletal muscle in transgenic mice compared with wild-type mice (Joe et al., 2012b). By overexpressing myocilin in transgenic mice, several protein complex components are redistributed and Akt, a key muscleizer, is phosphorylated (Joe et al., 2012a). Judge, Sarah M et al. identified MEF2C as a key upstream transcription factor required to maintain MyoC expression in skeletal muscle, MEF2C gain-of-function inhibits the downregulated of MyoC and prevents skeletal muscle wasting and dysfunction caused by cancer (Judge et al., 2020). The importance of MEF2C in cardiomyocyte hypertrophy has been well established (Feng et al., 2008; Papait et al., 2017; Zhao et al., 2021).

Studies have shown that local injection of the pro-inflammatory factor TNF-α can induce hyperalgesia in rats (Schäfers et al., 2003). Inflammation is a crucial feature of MTrPs (Carp et al., 2007; Shah et al., 2008; Grosman-Rimon et al., 2016). Our research group verified in previous unpublished studies that TNF-α was highly expressed in MTrPs. Under a light microscope, the typical feature of MTrPs were abnormally contracted muscle fibers (Zhang et al., 2020b; Jin et al., 2020; Jin et al., 2020). In this study, our group provides preliminary evidence for a pathogenic mechanism of MEF2C in skeletal muscle leading to pathological myofiber hypertrophy associated with chronic inflammation. We detected activation of the NF-κB signaling pathway in the gastrocnemius muscle of healthy rats after TNF-α injected and developed nociceptive hyperalgesia. And the injection of the NF-κB inhibitor Maslinic acid reversed nociceptive hypersensitivity suggesting that the NF-κB signaling pathway plays a key role in the inflammatory pain of MTrPs. Multiple inflammatory factors can activate the NF-κB signaling pathway, and the activated NF-κB signaling pathway can in turn promote the expression of multiple inflammatory factors (Lawrence, 2009; Sun, 2017). Activated NF-κB p65 undergoes nuclear translocation and binds to the promoter of MLCK to amplify MLCK, the mechanism that has been well studied in intestinal smooth muscle (Wang et al., 2021). After injection of Maslinic acid, the expression levels of MLCK exhibited the same tendency as the NF-κB p65. In the present study, we found that there is also a mechanism of NF-kB regulation of MLCK in skeletal muscle. MLCK leads to p300/PCAF recruitment by directly phosphorylating MEF2C, increasing acetylation of skeletal muscle-specific genes, and enhancing skeletal myogenesis (Al Madhoun et al., 2011).

**FIGURE 8:**
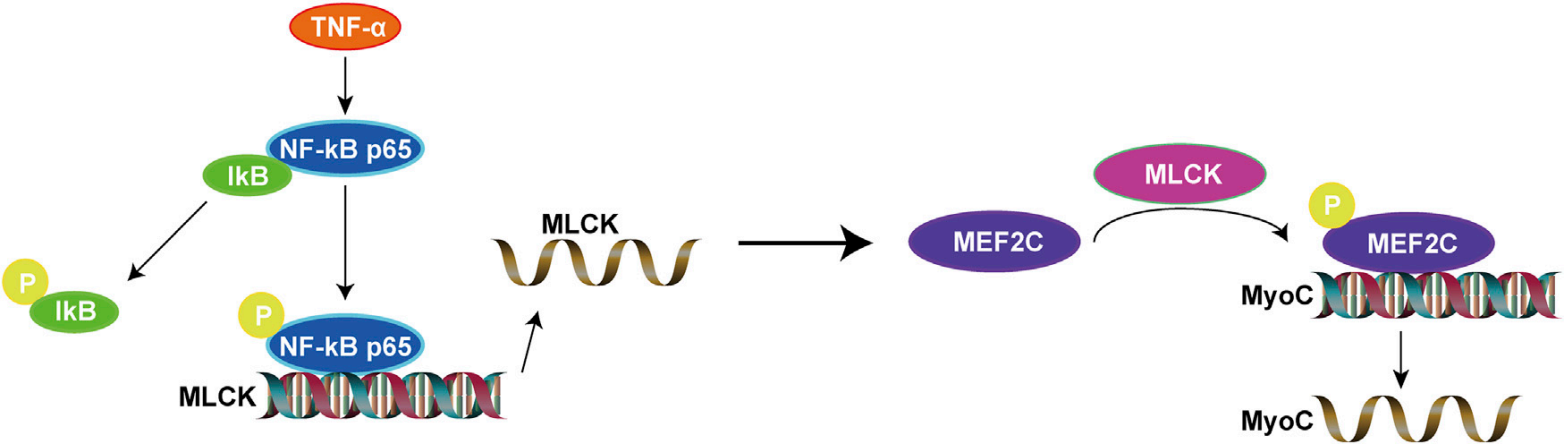
The proposed role of MEF2C in the inflammatory pathogenesis of MTrPs.

In this study, overall, we found that upregulated MEF2C is due to the activation of NF-κB signaling pathway. In contrast, inhibition of NF-κB signaling pathway can suppress MEF2C expression. This makes clear the upstream and downstream relationships involved. Overexpression of TNF-α in healthy rats increases MEF2C and activates the NF-κB signaling pathway, which is a classical inflammatory signaling pathway. Considering the results above, we propose the perspective that inflammatory cytokine (TNF-α) regulates the expression of MEF2C *via* the NF-κB/MLCK signaling pathway and in turn mediates inflammatory muscle fibers hypertrophy in MTrPs. Our results offer a novel perspective on abnormal muscle fibers hypertrophy and contribute to our understanding of the pathophysiological process of MTrPs (Figure 8).

Furthermore, the main methods currently used for local injection treatment of MTrPs are botulinum toxin, local anesthetics, dry needling, etc. Several clinical studies of botulinum toxin injections for the treatment of MTrPs have shown that the effects remain controversial (Ho and Tan, 2007; Gerwin, 2012; Soares et al., 2012). Dry needling can relieve pain in MTrPs in the short term, but its long-term therapeutic effects and relieving the dysfunction of MTrPs are still debatable (Liu et al., 2015; Pérez-Palomares et al., 2017; Liu et al., 2018). And, it has been confirmed that ultrasound-guided local anesthetic drug injections of MTrPs are less effective than ultrasound-guided dry needling, but the pain-relieving effects are obvious (Diep et al., 2021). The anti-inflammatory effect of DEX and its inhibition of NF-κB have been well studied (Wang et al., 2017; Wang et al., 2019; He et al., 2021). However, there are no studies on the use of DEX for the treatment of MTrPs. In this study, the phosphorylation of the NF-κB signaling pathway and the expression of MEF2C were inhibited after injection of DEX in the rat gastrocnemius muscles. More importantly, study results demonstrated that local injection administration was an effective pathway by comparing tail vein administration to local injection administration. These results illustrate that DEX has the potential to be used in the clinical treatment of MTrPs, but further clinical trials are needed to examine this possibility.

## Data Availability

The original contributions presented in the study are included in the article/Supplementary Material, further inquiries can be directed to the corresponding authors.

## Abbreviations

DEX, Dexmedetomidine; M.A., Maslinic acid; MPS, Myofascial pain syndrome; MTrPs, Myofascial trigger points; MEF2C, Myocyte enhancer factor 2C; TB, Taut band.
